# Identification and Characterization of Inhibitors of Human Apurinic/apyrimidinic Endonuclease APE1

**DOI:** 10.1371/journal.pone.0005740

**Published:** 2009-06-01

**Authors:** Anton Simeonov, Avanti Kulkarni, Dorjbal Dorjsuren, Ajit Jadhav, Min Shen, Daniel R. McNeill, Christopher P. Austin, David M. Wilson

**Affiliations:** 1 NIH Chemical Genomics Center, National Human Genome Research Institute, National Institutes of Health, Bethesda, Maryland, United States of America; 2 Laboratory of Molecular Gerontology, National Institute on Aging, National Institutes of Health, Baltimore, Maryland, United States of America; University of Minnesota, United States of America

## Abstract

APE1 is the major nuclease for excising abasic (AP) sites and particular 3′-obstructive termini from DNA, and is an integral participant in the base excision repair (BER) pathway. BER capacity plays a prominent role in dictating responsiveness to agents that generate oxidative or alkylation DNA damage, as well as certain chain-terminating nucleoside analogs and 5-fluorouracil. We describe within the development of a robust, 1536-well automated screening assay that employs a deoxyoligonucleotide substrate operating in the red-shifted fluorescence spectral region to identify APE1 endonuclease inhibitors. This AP site incision assay was used in a titration-based high-throughput screen of the Library of Pharmacologically Active Compounds (LOPAC^1280^), a collection of well-characterized, drug-like molecules representing all major target classes. Prioritized hits were authenticated and characterized via two high-throughput screening assays – a Thiazole Orange fluorophore-DNA displacement test and an *E. coli* endonuclease IV counterscreen – and a conventional, gel-based radiotracer incision assay. The top, validated compounds, i.e. 6-hydroxy-DL-DOPA, Reactive Blue 2 and myricetin, were shown to inhibit AP site cleavage activity of whole cell protein extracts from HEK 293T and HeLa cell lines, and to enhance the cytotoxic and genotoxic potency of the alkylating agent methylmethane sulfonate. The studies herein report on the identification of novel, small molecule APE1-targeted bioactive inhibitor probes, which represent initial chemotypes towards the development of potential pharmaceuticals.

## Introduction

Most drugs employed to eradicate neoplastic disease (e.g. alkylators, cross-linking agents, topoisomerase inhibitors and certain antimetabolites) operate by introducing DNA lesions/intermediates that block replication of rapidly dividing cells, such as cancer cells, and activate cell death responses [1]. Alkylators, for instance, induce cell killing through the formation of alkylated bases, many of which are either lost spontaneously to form abasic sites or are substrates for DNA glycosylases [2] (see below). A primary goal of current studies is to devise combinatorial methods that (a) protect normal cells from the toxic effects of anti-cancer agents and (b) enhance the sensitivity of tumor cells. As DNA repair systems represent a major protective mechanism against the cytotoxic effects of clinical DNA-interactive compounds, recent efforts have focused on the design of novel small molecule inhibitors of DNA repair proteins, e.g. the DNA strand break response protein poly(ADP)ribose polymerase PARP1 [3], [4].

BER is the major pathway for dealing with spontaneous hydrolytic, oxidative and alkylative base and sugar damage to DNA [5]. Central to this process is incision at an apurinic/apyrimidinic (AP) site, which is generated either spontaneously or via the enzymatic activity of a DNA repair glycosylase. The ensuing strand cleavage step is performed by the main, if not sole, mammalian AP endonuclease, APE1 [6], [7]. Significantly, APE1 has been found to be essential for not only animal viability, but also for cell viability in culture [8], [9]. Moreover, past studies incorporating either antisense or RNAi strategies have revealed that APE1-deficient cells exhibit hypersensitivity to a number of “DNA-damaging” agents, including the laboratory chemicals methyl methanesulfonate (MMS) and hydrogen peroxide, and the anticancer agents ionizing radiation, thiotepa, carmustine, temozolomide, gemcitabine, and the nucleoside analogue troxacitabine [10]. Recent work from our group employing a dominant-negative APE1 protein (termed ED), which binds with high affinity to substrate DNA and blocks subsequent repair steps [11], has shown that ED augments the cell killing of 5-fluorouracil and 5-fluorodeoxyuridine, implicating BER in the cellular response to such antimetabolites as well (McNeill et al., in press) [12]. These data underscore the potential of APE1 as a target for inhibition in the effort to improve therapeutic efficacy of clinical DNA-interactive drugs via inactivation of DNA repair responses [1].

Two groups have recently reported on the isolation of APE1 inhibitors using a high-throughput screening (HTS) approach. However, in the first instance [13], the reported effectiveness of this compound (i.e. CRT0044876 or 7-nitro-1H-indole-2-carboxylic acid) has not been reproduced [14]. In the second case, the small molecules (i.e. arylstibonic acids) when used in culture did not elicit a cellular outcome typical of APE1 inactivation, such as increased sensitivity to the alkylating agent MMS [15]. Furthermore, antimony-containing compounds are generally considered undesirable from a probe development standpoint due to their possible promiscuity akin to the effect of heavy metal ions and their occasional high toxicity [16]. Thus, there is a need for improved biochemical, and effective biological, inhibitors of APE1. BER inhibitors or activators would provide novel resources, not only for basic science purposes, but for the potential development of high affinity targeted, therapeutics that may improve the efficacy of treatment paradigms by promoting selective sensitization of diseased cells or increasing the protection of normal cells, respectively.

## Methods

### Reagents

Thiazole Orange (ThO), Tris-HCl, Tween-20, EDTA, NaCl, MgCl_2_ and dithiothreitol (DTT) were purchased from Sigma–Aldrich. Dimethyl sulfoxide (DMSO, certified ACS grade) and arylstibonic inhibitors (NSC-13744, NSC-13793, NSC-15596, and NSC-13755) were obtained from Fisher, Inc. and the National Cancer Institute Developmental Therapeutics Program Natural Products Repository, respectively. Black solid-bottom 384-well and 1536-well plates were purchased from Greiner Bio One (Monroe, NC).

### Compound library

The Sigma-Aldrich Library of Pharmacologically Active Compounds (LOPAC^1280^) were received as 10 mM DMSO stock solutions and were arrayed for screening as plate-to-plate (vertical) dilutions at 5 µL each in 1536-well Greiner polypropylene compound plates by following previously published methods [17], [18].

### Enzymes and fluorogenic substrates

**Figure 1:**
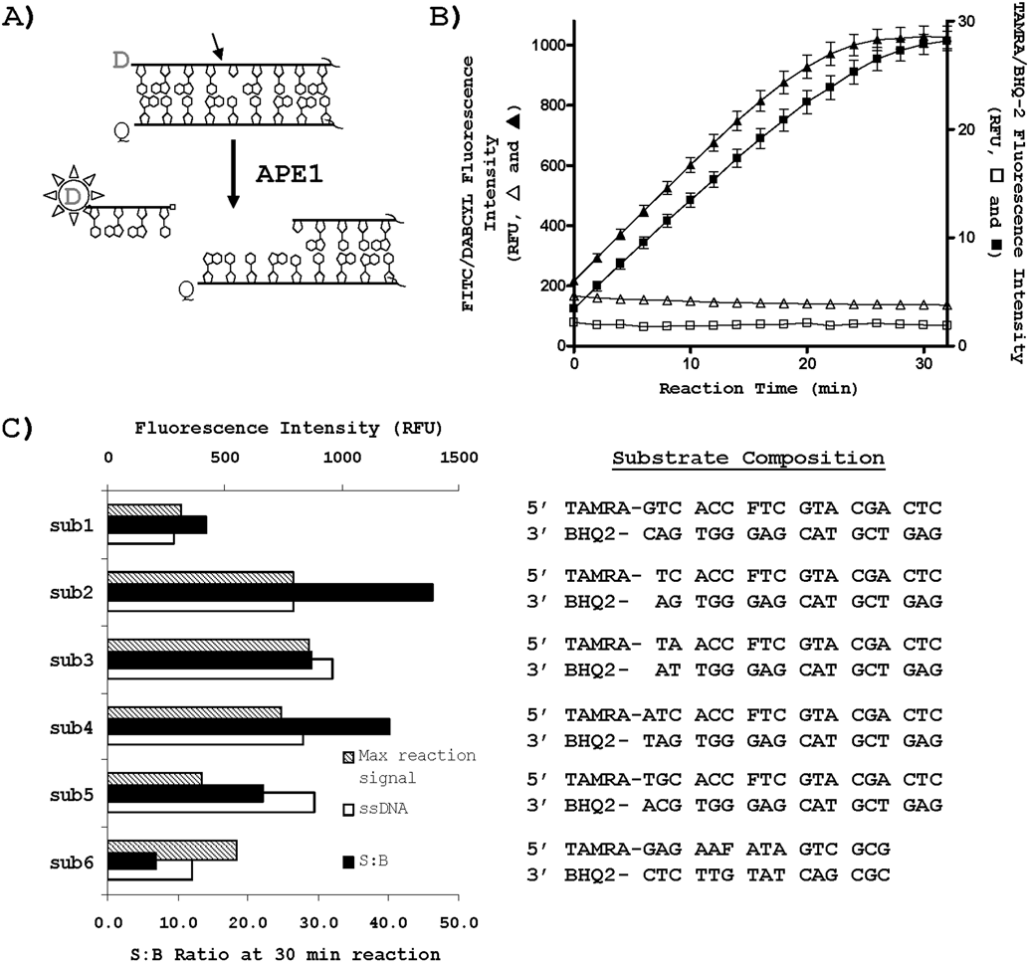
Incision assay. A) APE1 incises 5′ to the abasic site analog (THF) to liberate a short 5′-fluorophore donor (D)-labeled deoxyoligonucleotide, causing increased fluorescence signal. D can be any fluorophore, and Q represents any compatible quench moiety. The APE1 incision site is indicated by the arrow. The right side of the duplex is not complete, as denoted by the squiggly lines. B) Comparison of assay performance for green (triangles) and red (squares) substrates. Kinetic time course assay was run at room temperature at 50 nM substrate in duplicate with (filled symbols) or without (empty symbols) 0.75 nM APE1. The nucleotide sequence of the duplex substrates is in panel C, sub1, with the 5′ fluorophore and 3′ quench incorporated as indicated. C) Sequence optimization of red-shifted substrates. Shown are the fold signal changes after a 30 min reaction (S∶B ratio), the maximum raw-fluorescence reaction signals obtained from 50 nM substrate incubated with 0.75 nM APE1, and the raw-fluorescence signals observed from 50 nM of single-stranded TAMRA-labeled deoxyoligonucleotide (ssDNA, unquenched strand). The nucleotide composition of sub1 through sub6 is indicated to the right. Note that sub6 is identical to that employed by Stivers [15].

Recombinant human APE1 and *E. coli* EndoIV were purified as previously described [19]. Deoxyoligonucleotides containing a tetrahydrofuran (THF) AP site analog, carboxytetramethyl rhodamine (TAMRA), or Black Hole Quencher-2 (BHQ-2), as appropriate (see Figure 1), were purchased from Biosearch Technologies, Inc., (Novato, CA). To create double-stranded DNA substrates, equal volumes of 200 µM solutions of the respective strands in reaction buffer were mixed and incubated at 95°C for 5 min. The annealing mixture was allowed to cool gradually to room temperature. Annealing and assay experiments were performed in the following buffer: 50 mM Tris pH 7.5, 25 mM NaCl, 2 mM MgCl_2_, 1 mM DTT, 0.01% Tween-20.

### Assay development and optimization

Initial optimizations were carried out in 384-well format at a 40 µL total reaction volume. Briefly, 30 µL of APE1 were pipetted into the plate and incubated at room temperature for 10 min; subsequently, 10 µL of substrate were added to start the reaction. The final assay concentrations of the enzyme and substrate were 0.75 nM and 50 nM, respectively. Kinetic fluorescence data were collected on a ViewLux high-throughput CCD imager (Perkin Elmer, Waltham, MA) equipped with standard FITC (excitation filter 480 nm and emission filter 530 nm) or BODIPY (excitation filter 525 nm and emission filter 598 nm) optics.

### qHTS protocol and data analysis

Three µl of reagents (buffer in columns 3 and 4 as negative control and APE1 in columns 1, 2, 5–48) were dispensed into a 1536-well plate. Compounds (DMSO solutions) and control (DMSO only) (23 nL) were transferred via Kalypsys pintool equipped with 1536-pin array. The plate was incubated for 15 min at room temperature, and then 1 µL of substrate (50 nM final concentration) was added to start the reaction. Library plates were screened from the lowest to highest concentration.

Screening data were corrected and normalized, and concentration–effect relationships were derived by using in-house developed, publicly available algorithms (http://www.ncgc.nih.gov/pub/openhts/curvefit/). Percent activity was computed after normalization against the uninhibited, or neutral, control (64 wells, entire columns 1 and 2) and the no-enzyme, or 100% inhibited, control (64 wells, entire columns 3 and 4), respectively. A four parameter Hill equation was fitted to the concentration-response data by minimizing the residual error between the modeled and observed responses.

### Radiotracer incision assay

The radiotracer assay was performed essentially as described [20]. In brief, prioritized candidate inhibitor compounds from the qHTS assay were incubated at various concentrations (100 µM data shown) with APE1 (100 pg) at room temperature for 15 min in 25 mM Tris pH 7.5, 25 mM NaCl, 1 mM MgCl_2_, 1 mM DTT and 0.01% Tween-20. At that time, 0.5 pmol ^32^P-radiolabeled THF-containing 34 mer double-stranded DNA substrate [21] was added. Incision reactions were then carried out immediately at 37°C for 10 min in a final volume of 10 µL. After the addition of an equal volume of stop buffer (0.05% bromophenol blue and xylene cynol, 20 mM EDTA, 95% formamide), the radiolabeled substrate and product were separated on a standard polyacrylamide denaturing gel and quantified by phosphorimager analysis [21].

### ThO dye-displacement assay [22]

During initial optimization, 50 nM of the unlabeled version of the red substrate (Figure 1) was titrated with ThO in 40 µL in 384-well format; 250 nM ThO was selected for subsequent tests. For compound profiling, the assay was miniaturized to 4 µL in a 1536-well plate by direct volume reduction. Compounds were added to a 4 µL mixture of 50 nM DNA and 250 nM ThO via pintool transfer and the fluorescence signal (excitation 480 nm, emission 530 nm) was measured after a 15 min incubation at room temperature.

### *E.coli* Endonuclease IV (EndoIV) profiling assay

EndoIV enzyme and substrate (same as that used with APE1) optimization tests were initially performed at a 40 µL final volume in a 384-well format. Reaction concentrations of 2 nM EndoIV and 50 nM DNA substrate were selected for subsequent experiments. For running the assay in 1536-well format, the same protocol as described for APE1 above was followed.

### Whole cell extract assay

HEK 293T or HeLa cells – maintained in DMEM with 10% fetal bovine serum and 1% Penicillin-Streptomycin – were harvested, washed with 1× PBS, re-suspended in cold 222 mM KCl, incubated on ice for 30 min, and clarified by centrifugation at 12,000×g for 15 min at 4°C [23]. The supernatant (whole cell extract) was retained, the protein concentration determined using the Bio-Rad Bradford reagent, and aliquots were stored at −80°C. AP endonuclease activity assays were performed with or without candidate inhibitor compounds as described above, except 60 ng HEK 293T or 80 ng of HeLa whole cell extract was used.

### Cell-based survival assay

To measure colony survival, HeLa cells were plated in 6-well plates 12–20 hr prior to treatment. Cells were treated as follows: (i) exposed only to control medium, (ii) exposed to 0.25 mM MMS alone for 1 hr, (iii) exposed to 1 or 5 µM of the indicated inhibitor compound alone for 4 hr, or (iii) exposed to inhibitor compound alone for 3 hr and then with MMS for an additional 1 hr at 37°C. Following treatment, cells were washed once with 1× PBS and incubated in regular maintenance media (DMEM, 10% FBS) at 37°C for 10–15 days. Colonies formed were fixed in methanol, stained in methylene blue (Sigma), and counted to determine percent survival relative to the untreated controls.

### AP site measurement

HeLa cells were treated as above, except only at 5 µM inhibitor where indicated. Following exposure, chromosomal DNA was isolated and steady-state AP site levels were measured using the DNA Damage Quantification Kit from Dojindo Molecular Technologies, Inc. (Gaithersburg, MD) as previously described [11].

### Molecular modeling

The preparation of the ligand for docking was performed using OMEGA version 2.3.1, part of the OpenEye Scientific Software suite (http://www.eyesopen.com/). Multiple low-energy conformations of the ligands were generated and partial charges were assigned to the ligand using MMFF94 force field [24]. The RMS threshold between different conformers of OMEGA was set to 0.5 Å. A total of 137 conformers were generated for DL-DOPA. DNA-bound crystallographic structure of APE1 was prepared for the docking studies using MOE molecular modeling software (http://www.chemcomp.com/). DNA strand has been cleaved at the phosphodiester bond to leave sugar-phosphate backbone at a position 5′ of AP active site as the reference ligand for docking. All hydrogens were added to the protein and partial charges were attributed to the protein atoms using Amber99 force field [25].

The active site of APE1 in which the sugar phosphate binds was characterized using FRED docking preparation program fred_receptor (http://www.eyesopen.com/) to generate optimal binding pose within the active site defined by the user. First, during an exhaustive docking, a pose ensemble is generated by rigidly rotating and translating each conformer within the active site. All surviving poses are scored with a scoring function (Chemgauss3) and the top 100 poses are passed to optimization. Next, a systematic solid body optimization is done by rigidly rotating and translating the poses at half the step size used in the exhaustive docking. Chemgauss3 is used in this step to score the poses during optimization. Lastly, poses are ranked via consensus structure method, in which the poses with the top consensus scores of Shapegauss, PLP, Chemgauss2 and Chemgauss3 are retained, and all other poses are discarded. Optionally after consensus scoring, poses can be refined using the Merck Molecular Mechanics Force Field. The refinement consists of full coordinate optimization of all ligand atoms, and any poses that violate the constraints are discarded after the refinement.

## Results

### AP endonuclease assay design and optimization

The starting point for our assay was the recently published donor/quencher-based approach described by the laboratories of Hickson and Stivers [13], [15]. In their experiments, a deoxyoligonucleotide containing an internal THF abasic site analog and a 5′ 6-carboxyfluorescein (FAM) label was annealed to a complementary strand with a 3′ DABCYL quencher to create a double-stranded DNA substrate. The close proximity of the fluorophore and the quencher results in a dampened signal upon light excitation. Following DNA backbone cleavage by APE1 [20], a short deoxyoligonucleotide fluorophore-labeled product is spontaneously released from the remaining DNA fragment possessing the quencher, causing the fluorophore emission to increase (Figure 1A).

However, the FAM/DABCYL format suffers from compound fluorescent interference, which is readily evident in the fluorescein (hereafter referred to as “green”) spectral region. Therefore, we proceeded to test red-shifted APE1 substrates (hereafter referred to as “red” substrates) by utilizing a combination of carboxytetramethyl rhodamine (i.e. TAMRA) as the fluorophore donor and BHQ-2 [26] as the matching quencher. This arrangement operates in the red-shifted spectral region, where very few compound library members have been noted to fluoresce [27]. As evident from Figure 1B, the red substrate exhibited near-identical cleavage kinetics with APE1, but provided an approximately twofold better signal increase, in comparison with the green version.

APE1 exhibits little substrate sequence specificity [28], as it must operate on abasic sites throughout the genome. Since we had the ability to modify the nucleotide sequence at will, we designed and tested several variations of the THF-containing red substrate to identify the optimal arrangement for the screening assay. In total, six substrates were examined, with their designations (sub1 through sub6) and composition indicated in Figure 1C. These substrates were compared to a red-shifted version (sub6, Figure 1C) of the previously published green substrate of Stivers [15]. The fold signal changes after a 30 min reaction, as well as the maximum reaction signals and the signal at 50 nM of single-stranded TAMRA-labeled deoxyoligonucleotide, were compared. Substrate 2 exhibited the greatest signal increase as a result of APE1 cleavage, likely due to its optimal G/C content for maximal quenching of the fluorophore and 5′ length for dissociation after incision (Figure 1C), and was thus selected for all subsequent studies. We note that substrate 6 yielded the smallest signal increase, mainly due to a higher baseline signal that likely arose from “wobbling” of the shorter and more AT-rich upstream portion.

Red substrate 2 (hereafter referred to as substrate) was further evaluated in 384-well plates by recording the initial reaction rates as a function of substrate concentration. From these studies (data not shown), a Km value of 65 nM was estimated. The similarity in Km obtained for substrate 2 here with the values reported previously using a traditional radiolabeled substrate [29] served as an additional validation of the present fluorogenic red-shifted format. A substrate concentration of 50 nM was chosen for the subsequent quantitative HTS (qHTS) experiments.

### qHTS of the Library of Pharmacologically Active Compounds (LOPAC^1280^)

After further assay miniaturization to a 4 µL volume in a 1536-well format (see Methods), a set of recently-reported known APE1 inhibitors, i.e. the arylstibonic acid derivatives [15], were obtained from the National Cancer Institute and tested as controls; robust concentration-dependent, fluorophore-independent inhibition was observed (Figure S1). In addition, the IC_50_ values found here were similar to the values previously obtained for the arylstibonic acid derivatives [15] despite the differences in assay conditions (enzyme and magnesium concentration, as well as substrate sequence disparity), further verifying the functionality of our approach. Figure S2 demonstrates that the assay reagents, as formulated at their screening concentrations, were stable over a 24 hr period. This stability, coupled with the robust assay performance in the 1536-well plate format, indicates that the enzyme is screenable in an automated, unattended fashion.

**Figure 2:**
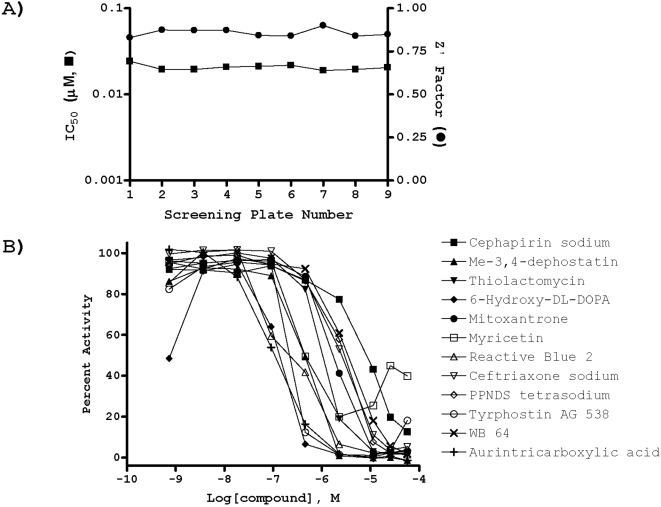
LOPAC^1280^ qHTS. A) Robust screen performance as evidenced by the reproducible, high Z' factor as a function of screening plate number (solid squares) and stable IC_50_ values for the arylstibonic derivative NSC-13755 used as a control in each screening plate (solid circles). B) Examples of representative concentration-response curves associated with a set of actives (denoted) identified in the screen.

The optimized APE1 assay was utilized in a qHTS of the LOPAC^1280^, which is composed of a range of drug-like bioactive molecules representing all major target classes. The library was screened in a dose response format at compound concentrations ranging from 2 nM to 57 µM [17], [18], [30]. At the end of the screen, data were analyzed and an average Z' factor of 0.86 was determined. Importantly, the Z' factor [31] remained essentially constant throughout the screen (Figure 2A), supportive of a highly stable assay. In addition, control titrations with the arylstibonic acid derivative NSC-13755 (see Figure S1), which were present in column 2 of every screening plate, produced high-quality, concentration-response curves, with the associated IC_50_ remaining nearly constant as the screen progressed (Figure 2A). The minimum significant ratio (MSR) [32] was 1.30, further corroborating a stable run.

The screen yielded a wide range of inhibitor responses (see PubChem BioAssay Summary, AID 1705). Twelve compounds were characterized by complete concentration-response curves and IC_50_ values better than 5 µM (Figure 2B). Additionally, 44 samples yielded incomplete, single-point, top-concentration inhibitory responses. A large fraction of these latter compounds were noted to possess hydrophobic functionalities, which are frequently associated with propensity to aggregate or precipitate; these hits received a low priority going forward. Only one strongly fluorescent hit, idamycin, also known as idarubicin [33], was noted.

**Figure 3:**
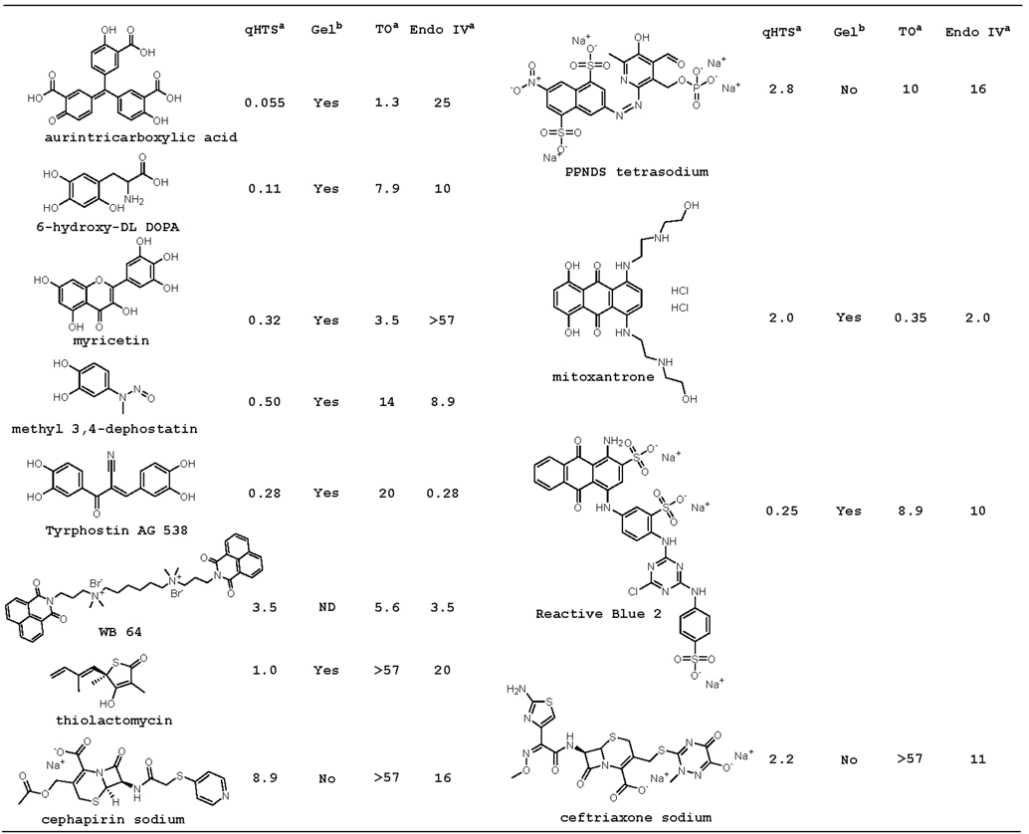
Top screening hits for APE1. The activities of the compounds shown (^a^, IC_50_ in µM, ^b^, inhibition detected (Yes) or not detected (No)) are given for the primary screen (qHTS), radiotracer incision assay (Gel), ThO dye-displacement assay (TO), and *E. coli* EndoIV profiling (EndoIV). ND, not determined. Me = methyl.

Aurintricarboxylic acid (ATA) was identified as the most potent inhibitor of APE1 in the LOPAC collection (Figure 2B). Additional hits included a wide variety of small molecule bioactives: cephalosporin type antibiotics (cephapirin sodium and ceftriaxone sodium), the wide-acting flavonoid myricetin, the purinoceptor antagonist Reactive Blue 2, the DNA-binding anticancer drug mitoxantrone, the nitrosoaniline type protein tyrosine phosphatase inhibitor methyl-3,4-dephostatin [34], the antimycobacterial and antitrypanosomal fatty acid synthesis inhibitor thiolactomycin [35], and the dopamine precursor 6-hydroxy-DL-DOPA. Figure 3 shows the chemical structures of the candidate APE1 inhibitors, prioritized based on the following criteria: low or sub µM IC_50_ value, chemical diversity, and not likely fluorescent. Top active compounds were confirmed by re-testing of independently procured powder samples in the HTS assay and subjected to the investigations described next.

### Validation and profiling assays

#### DNA binding

**Figure 4:**
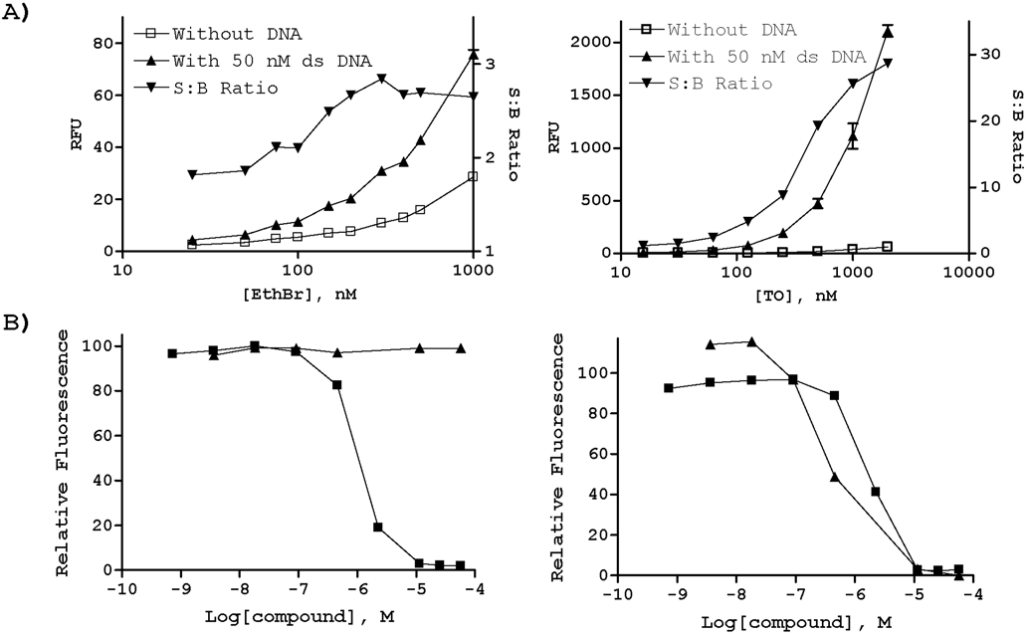
ThO displacement assay. A) Assay optimization using either ethidium bromide (EthBr, left) or ThO (right) as the DNA bound fluorophore. Plotted are raw fluorescence signal from duplicate wells (RFU), as well as S∶B, computed as the signal ratio from DNA-containing (50 nM ds DNA) versus buffer-only (i.e. without DNA) wells. B) Shown as examples are the effects of thiolactomycin (left) and mitoxantrone (right) on ThO displacement (filled triangles, fluorescence signal normalized against DNA-free (0% relative fluorescence) and DNA-containing (100% relative fluorescence) controls) or APE1 incision activity in the screening assay (filled squares).

To screen out compounds that inhibit APE1 activity via non-specific DNA binding, we employed a fluorescent dye displacement assay essentially as described by Boger [22]. The principle behind the assay is that if a compound acts non-specifically by associating with DNA, it will displace a DNA bound flourophore, consequently decreasing the signal reading of the sample. In order to arrive at the most optimal assay with respect to the signal window and minimal interference from compound autofluorescence, we compared the performance of ethidium bromide, the dye most commonly used in this assay, to ThO, in side-by-side titration experiments using an unlabeled version of the optimized APE1 substrate (Figure 1C). ThO, which binds non-covalently to DNA with high affinity, was selected as the displacement dye for the studies herein (see below), because it was found to offer superior signal (Figure 4A) and because its fluorescence excitation and emission are red-shifted relative to those of ethidium bromide, thus ensuring reduced susceptibility to compound autofluorescence.

The ThO displacement assay was used to profile the LOPAC^1280^ collection in a dose-response format (see PubChem BioAssay Summary, AID 1707). The well-documented DNA-binding anticancer agents, idarubicin and doxorubicin, exhibited strong ThO displacement profiles (data not shown), validating the utility of the approach. Among the top APE1-active compounds identified in the initial HTS, WB 64 and mitoxantrone (representative plot shown in Figure 4B, right) were positive in the DNA-binding assay, suggesting that they likely act as non-competitive inhibitors (summarized in Figure 3). Thiolactomycin is shown in Figure 4B (left) as an example of a compound that inhibits APE1 activity, but does not affect ThO displacement.

#### EndoIV profiling screen

As another means of probing the selectivity of the candidate APE1 inhibitors (Figure 3), we miniaturized to a 1536-well format a qHTS assay for *E. coli* EndoIV [19] utilizing the same THF-containing red substrate as above (Figure S3). *E. coli* EndoIV, while exhibiting similar biochemical activities to APE1, such as AP site incision, has no sequence or structural homology to the human protein [36], and thus, serves as a powerful protein counterscreen to identify APE1 specific affectors. A qHTS of the LOPAC^1280^ collection (see Methods) revealed considerably fewer inhibitors for the bacterial enzyme (see PubChem BioAssay Summary, AID 1708). In fact, there were only 5 complete concentration-response curves for EndoIV, although as with APE1, a number of suspected aggregators or insoluble compounds displayed inhibition only at the top concentration. Furthermore, most of the EndoIV hits functioned at a significantly higher IC_50_ than seen for APE1.

**Figure 5:**
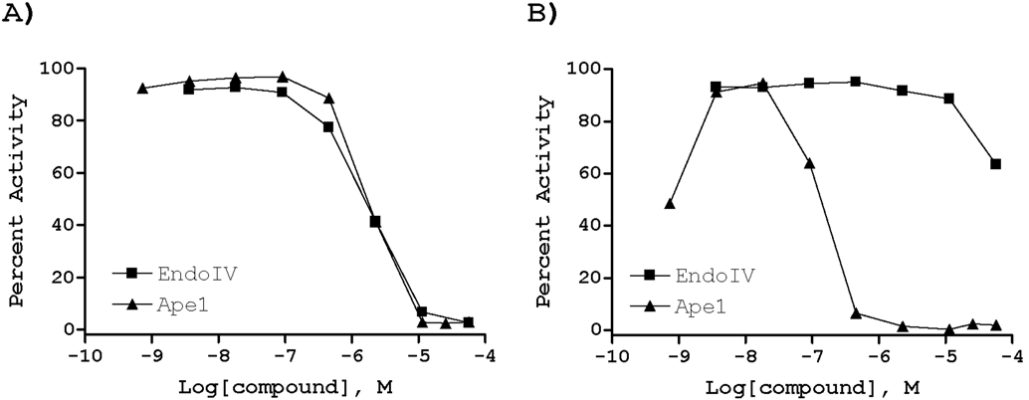
EndoIV counterscreen. Shown are examples of: (A, mitoxantrone) a molecule equally potent against APE1 and EndoIV and (B, 6-hydroxy-DL-DOPA) an inhibitor with an apparent APE1 selectivity.

Mitoxanthrone (representative plot shown in Figure 5), WB 64, Tyrphostin AG 538 and cephapirin sodium were similarly potent against each endonuclease (summarized in Figure 3). The uniform activity of mitoxantrone and WB 64 can be rationalized by their DNA binding affinity as determined in the ThO displacement assay (see above). Top APE1 hits, which were completely inactive or weakly active against EndoIV, included ATA, 6-hydroxy-DL-DOPA (representative plot shown in Figure 5), thiolactomycin, myricetin, methyl 3,4-dephostatin and Reactive Blue 2.

#### Radiotracer assay

**Figure 6:**
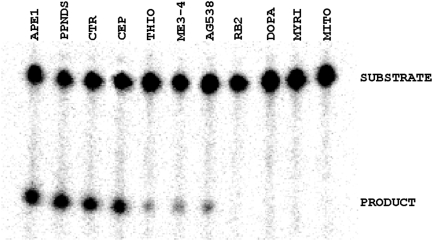
Radiotracer validation assay. APE1 incision of a ^32^P-labeled DNA substrate was measured following incubation with the indicated candidate inhibitor at 100 µM. Shown is a representative image of a denaturing polyacrylamide gel after resolution of the intact substrate from the smaller reaction products (denoted). APE1 = no inhibitor, DMSO control; PPNDS = PPNDS tetrasodium; CTR = ceftriaxone sodium; CEP = cephapirin sodium; THIO = thiolactomycin; ME3-4 = methyl-3,4-dephostatin; AG538 = Tyrphostin AG 538; RB2 = Reactive Blue 2; DOPA = 6-hydroxy-DL-DOPA; MYRI = myricetin; MITO = mitoxantrone.

As a means of further validating the initial APE1 inhibitors (Figure 3), we simultaneously determined the effect of the top actives on APE1 incision activity using a ^32^P-based oligonucleotide radiotracer assay (see Methods). The top APE1 hits were tested initially at 100 µM (Figure 6) and later by determining the approximate IC_50_ values using a range of inhibitor concentrations (data not shown). The extent of strand cleavage inhibition as determined in the radiotracer assay generally tracked that observed in the fluorogenic screening approach (as judged by the respective IC_50_ values): myricetin, mitoxanthrone, 6-hydroxy-DL-DOPA and Reactive Blue 2 showed near complete inhibition (IC_50_<0.5 µM); thiolactomycin, methyl 3,4-dephostatin and Tyrphostin AG 538 yielded an intermediate effect (IC_50_ in the 0.5–2 µM range); and PPNDS, cephapirin sodium and ceftriaxone sodium were poorly active (IC_50_>50 µM).

Taking into account the compilation of the results from the experiments above, we excluded from further analysis mitoxantrone, because the compound operated largely as a DNA binder and non-competitive inhibitor, and ATA, as the effect of this agent likely stems from its ability to act as a DNA mimic and direct competitor of nucleic acid processing enzymes in general [37]. We also eliminated from further characterization PPNDS, cephapirin sodium and ceftriaxone sodium, since they had at best weak, and seemingly non-specific, inhibitory effects (summarized in Figure 3). We performed more detailed analysis on myricetin, 6-hydroxy-DL-DOPA, Reactive Blue 2, thiolactomycin, methyl 3,4-dephostatin and Tyrphostin AG 538.

### Inactivation of AP endonuclease activity in whole cell extracts

**Figure 7:**
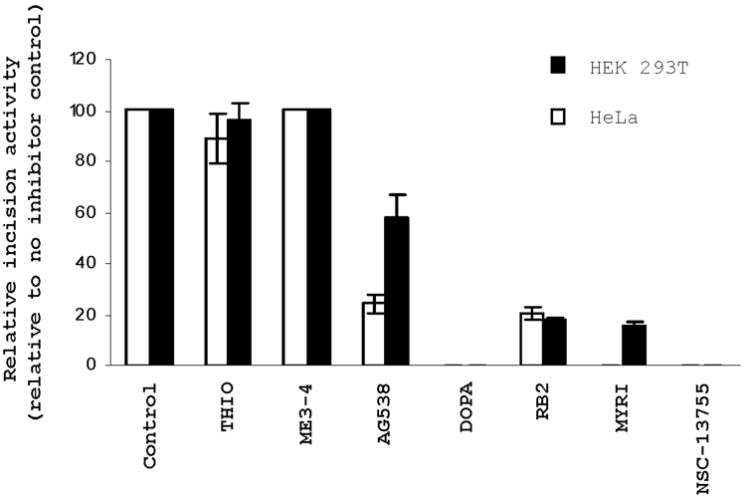
Effect of top APE1 inhibitors on AP site incision activity of whole cell extracts. Whole cell protein extracts from either HEK 293T or HeLa cells (denoted) were exposed to the indicated inhibitor at 100 µM prior to measuring DNA strand cleavage activity via the radiolabeled substrate assay. See Figure 7 for details and abbreviations; NSC-13755 = the arylstibonic derivative.

Since APE1 comprises >95% of the total AP endonuclease activity in mammals [38], most, if not all, THF incision observed in human whole cell extracts is the result of APE1-dependent cleavage. Thus, as another means of assessing the specificity of candidate APE1 inhibitors, and to begin to evaluate their potential biological value, we generated whole cell protein extracts from HEK 293T and HeLa cells and determined the effect of the most promising actives on total AP site cleavage activity [39]. Inhibitors with specificity for APE1 were expected to impart a reduced incision capacity relative to the control (no inhibitor, DMSO control) reaction, even amidst all the non-specific proteins in the extract. Indeed, 6-hydroxy-DL-DOPA and the arylstibonic acid derivative NSC-13755 (used largely as a control, but not previously tested in a whole cell extract assay) showed complete inhibition at 100 µM; Reactive Blue 2 and myricetin displayed potent inhibition (≥80%); and Tyrphostin AG 538 displayed mild inhibition against the HEK 293T and HeLa protein extracts (Figure 7). Thiolactomycin and methyl 3,4-dephostatin had no effect on total AP site cleavage activity of either extract.

### Enhancement of MMS cytotoxicity and genotoxicity

**Figure 8:**
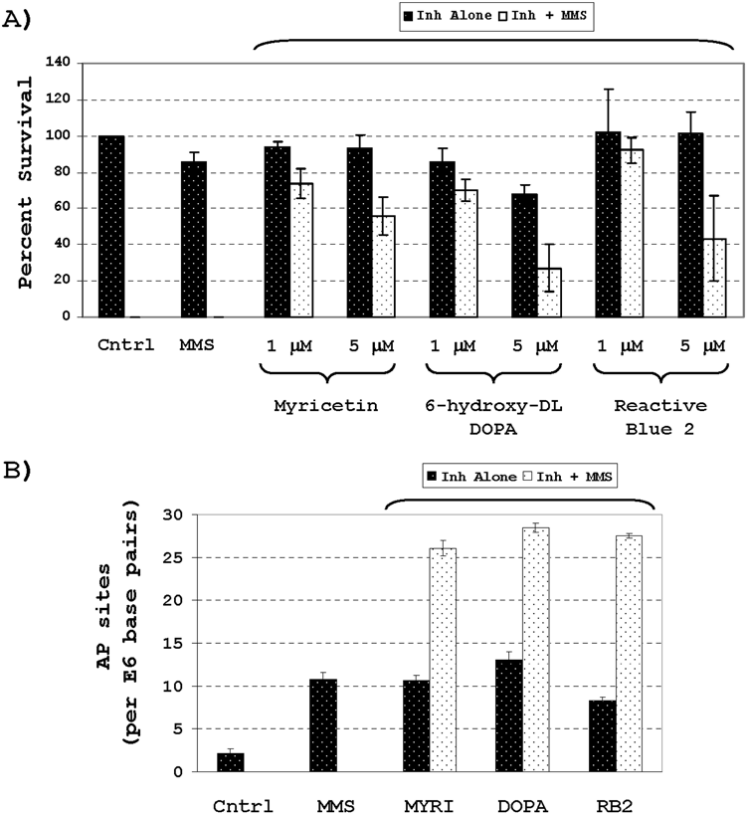
Effect of top APE1 inhibitors on cytotoxic and genotoxic potential of MMS. A) HeLa cells were exposed to control/mock conditions (cntrl), MMS alone, inhibitor (Inh) alone, or both inhibitor and MMS as indicated. Plotted is the average and standard deviation of the percent colony survival relative to the control for three independent data points. B) HeLa cells were exposed to the different conditions outlined in panel A (except only at 5 µM inhibitor), and total genomic AP sites were measured. Shown is the average and standard deviation of five independent measurements of the number of AP sites per 10^6^ base pairs.

As an additional means of examining the biological potential of the APE1 inhibitors displaying the most promise in the assays above, we evaluated the ability of 6-hydroxy-DL-DOPA, Reactive Blue 2 and myricetin to enhance cellular sensitivity to the alkylating agent MMS. MMS creates methylated base damage, which is either lost spontaneously or excised by the alkylpurine DNA glycosylase, resulting in a high number of cytotoxic abasic sites [2]. We compared the colony formation efficiency of HeLa cells exposed to MMS alone, one of the inhibitors alone, or a combination of MMS plus inhibitor relative to a non-exposed control. The combination of MMS and either of the inhibitors resulted in a greater than additive increase in the percent cell killing relative to the alkylator or inhibitor alone (at doses pre-determined to elicit minimal cytotoxicity; Figure 8A), suggesting a synergistic effect of these compounds on MMS lethality, as would be expected for APE1 inactivation [10].

As a more direct means of evaluating the effect of 6-hydroxy-DL-DOPA, Reactive Blue 2 and myricetin on APE1 activity *in vivo*, we measured the level of AP sites in chromosomal DNA from HeLa cells following one of the aforementioned treatment schemes using an established aldehyde reactive probe-based colorimetric assay [40]. These studies revealed that exposure to 6-hydroxy-DL-DOPA, Reactive Blue 2 or myricetin alone (at 5 µM) increased AP site damage relative to the control cells, and that each of the compounds potentiated the genotoxic potential of MMS, supporting targeted inactivation of APE1 repair function by these bioactives.

### Predicted binding of inhibitors in APE1 active site

**Figure 9:**
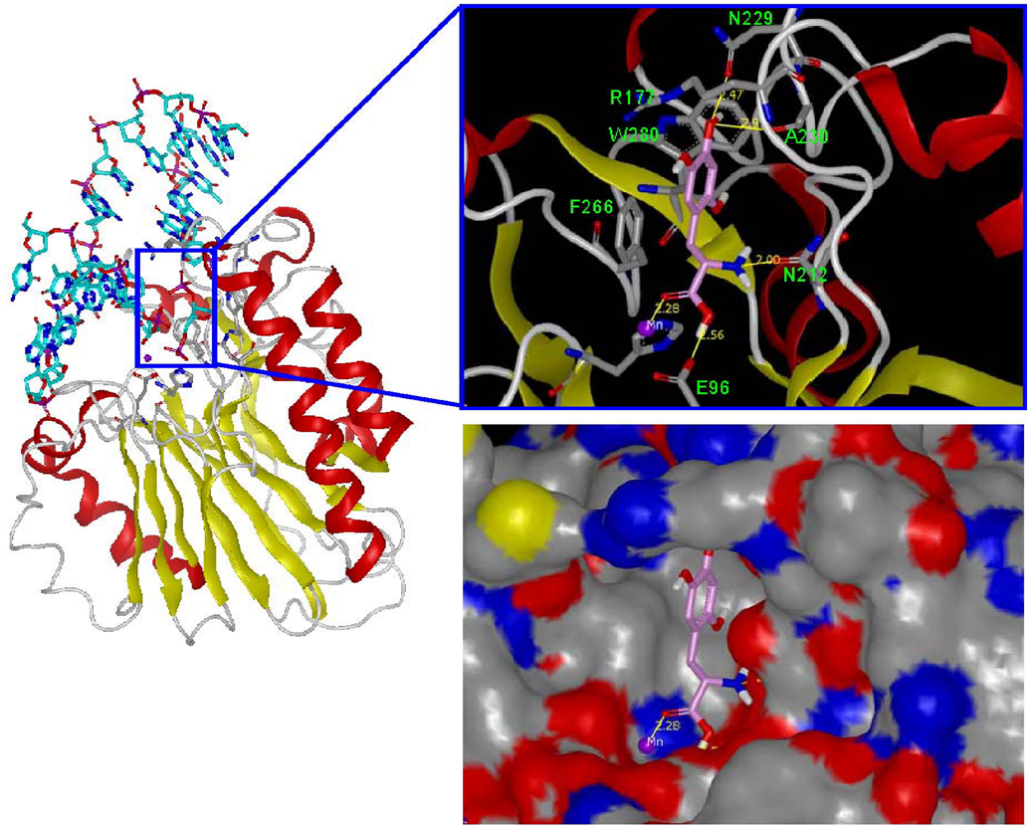
Molecular Modeling. Left panel: ribbon representation of APE1 structure with DNA bound (PDB code: 1DE9); DNA is shown in cyan; the active site is marked within the box. Upper right panel: docking pose of 6-hydroxy-DL-DOPA in APE1 active site; potential hydrogen-bonds are labeled in yellow lines with distances, selected active site amino acid residues are indicated. See text for details. Lower right panel: APE1 binding pocket is depicted by molecular surface representation.

Molecular docking enables the evaluation of preferred binding orientation and affinity of small molecules to their protein targets. In an attempt to understand the possible interaction modes of the top hits in the assays above, FRED docking was performed (see Methods). Using the available X-ray crystal structure of APE1 complexed with substrate DNA (PDB code: 1DE9), docking of 6-hydroxy-DL-DOPA into the APE1 binding site revealed that the sugar phosphate pocket can easily accommodate the aromatic moiety of the compound (Figure 9). The phenyl core fits in the hydrophobic pocket, which is bordered by active site residues Phe266, Trp280 and Leu282, known to complex with the abasic ring structure. The two oxygen atoms of the carboxylic acid moiety of 6-hydroxy-DL-DOPA are able to interact with both metal ion (Mn^2+^) and the catalytic residue Glu96 in a similar position to the corresponding DNA phosphate in the co-crystal structure. The 4-hydroxyl of the molecule can potentially hydrogen-bond with either the side chain oxygen of Asn229 or the backbone carbonyl oxygen of Ala230, while the primary amine is involved in a hydrogen bond with the side chain oxygen of Asn212. In addition, the phenyl ring of 6-hydroxy-DL-DOPA could form a parallel-displaced pi stacking interaction to Phe266 and an edge-to-face pi stacking to Trp280.

We also performed docking experiments on Reactive Blue 2 and myricetin; however, it was impossible to arrive at preferred binding orientations for these two compounds likely due to their structural features. Reactive Blue 2 is a bulky molecule containing multiple anchoring groups (sulfonic acids and aminotriazine), all of which could potentially interact with the metal ion in the APE1 active site, while myricetin is a rigid molecule lacking anchoring groups, resulting in numerous possibilities of docking orientations without shape complementarity conflicts.

## Discussion

Small molecule protein modulators are viewed as the perfect complement to frequently-used techniques such as RNAi and gene knockout. In addition, the scientific and medical communities have become increasingly interested in the design of small molecule inhibitors of DNA repair, with the potential of improving the therapeutic efficacy of clinical DNA-interactive drugs [1]. While there have been reports of small molecules directed at APE1 [10], [13], [14], [15], [39], [41], the utility of these inhibitors has been brought into doubt given (a) the inability to reproduce their effectiveness [14] (and our unpublished observations) or (b) the failure of the compounds to elicit a cellular consequence [15]. We describe herein (i) the development of a panel of complementary and improved miniaturized high-throughput screening and profiling assays, which we believe will have broad appeal to other investigators, and (ii) the identification of novel small molecule APE1 inhibitors, which represent initial chemotypes in the long-term endeavor of creating new targeted pharmaceuticals.

We outline within a robust APE1 screening assay that integrates TAMRA (a bright, pH-insensitive analogue of rhodamine) as the red-shifted fluorophore and BHQ-2 as the non-emitting dark quencher. While the FAM/DABCYL arrangement remains a convenient and largely appropriate choice in genotyping applications, its use in configuring small molecule screens is more problematic. In particular, in a recently performed profiling study, we found that up to 5% of the NIH Small Molecule Repository (MLSMR, https://mli.nih.gov/mli/mlsmr/) exhibited strong fluorescence in the blue-shifted UV light region (excitation near 350 nm, emission near 450 nm), and up to 0.2% of the diverse small molecule collection interfered with fluorescein-like detection (excitation near 480 nm, emission near 530 nm). Conversely, the incidence of compounds capable of interfering with detection in the red-shifted region (e.g, excitation around 550 nm and emission near 590 nm) diminished to below 0.01% [27]. Moreover, the combination of BHQ-2 with TAMRA resulted in a twofold improvement in the signal to noise ratio relative to the comparable FAM/DABCYL pair, and was better quenched in its dark state. We note that the implemented real-time fluorescence monitoring (kinetic read) to derive reaction rates also makes the TAMRA/BHQ-2 assay less susceptible to variations in the screening equipment and permits facile discovery of problematic compounds which may interfere with detection [30]. It is anticipated that the reconfigured APE1 assay described here will serve as a useful guide to future investigations aimed at screening other nucleic acid processing enzymes.

The concentration-response screen of the LOPAC collection yielded a number of previously-unreported APE1 inhibitors. The most potent hit was ATA, which inactivated the enzyme consistently in the low nanomolar range. While very effective, ATA has been noted to exist as a stable radical homopolymer of varying length and to act as a strong inhibitor of a large number of DNA- and RNA-processing enzymes [37], [42], [43]. As such, ATA did not represent a chemotype of value to study APE1 function. The other top hits (summarized in Figure 3) comprised a diverse group of compounds and included small molecules with potent inhibitory potential, such as 6-hydroxy-DL-DOPA, thiolactomycin and methyl 3,4-dephostatin, several larger-size comparatively weak inhibitors, such as PPNDS tetrasodium and ceftriaxone sodium, and suspected DNA binders, such as mitoxantrone and WB 64. Since a variety of factors, including promiscuous aggregators, non-selective covalent modifiers and compounds that sequester substrate molecules, can produce primary screening hits that are not relevant [44], we developed and implemented a panel of secondary assays to run against a subset of initial hits or the entire LOPAC collection.

As a means of interrogating the primary screening hits, and to gain further insight into their mechanism of action, we employed a fluorescent dye-displacement assay [22], substituting the frequently-used ethidium bromide with the more sensitive and robust reporter ThO. In a screen of the LOPAC collection against a ThO-substrate DNA complex, all annotated fluorescent DNA intercalators within the library, e.g. idarubicin, doxorubicin and distamycin, displayed strong displacement activity. Furthermore, the non-fluorescent APE1 screening hits WB 64 and mitoxantrone exhibited dye-displacement IC_50_ values similar to or better than those displayed in the APE1 enzymatic incision assay. This behavior is reminiscent of an indirect, non-competitive DNA binding effect and is consistent with the multiple fused ring systems, which have a tendency for DNA intercalation, featured in both molecules. On the other hand, APE1 inhibitor molecules that lacked obvious DNA-binding features (i.e. fused aromatic ring structures or positively charged groups), such as thiolactomycin and Tyrphostin AG 538, yielded weak or no displacement activity. These findings support the ThO displacement assay as a convenient counterscreen to exclude DNA binders from further consideration, and the complete results with the LOPAC^1280^ are available in the corresponding PubChem deposition (AID 1707).

To further probe the selectivity of the inhibitors identified in the APE1 qHTS, we tested the LOPAC collection against *E. coli* EndoIV. AP endonucleases are sub-classified into two major superfamilies based on homology to either *E. coli* exonuclease III (ExoIII) or *E. coli* EndoIV [36]. While members of the two superfamilies exhibit similar biochemical properties, such as AP endonuclease activity, there exists no sequence or structural homology between the different superfamily constituents. Since APE1 is an ExoIII family member, it was anticipated that inhibitors specific for human APE1 would not exert an effect on *E. coli* EndoIV. The screen of the LOPAC^1280^ against EndoIV under conditions identical to those used in the APE1 screen resulted in relatively few hits, most of which had low potency. Among the actives shared between the two endonucleases were the non-specific DNA binders mitoxantrone and WB 64, whereas compounds with selectivity to APE1 included thiolactomycin, 6-hydroxy-DL-DOPA, methyl 3,4-dephostatin, myricetin and Reactive Blue 2. The fact that the EndoIV screen did not identify either potent (other than Tyrphostin AG 538; Figure 3) or EndoIV-selective hits may suggest an active site for this enzyme that is hard to access. Our ongoing studies of inhibitors for *E. coli* EndoIV are outside the scope of the present investigation and will be the subject of a separate report.

Finally, we tested the top screening hits in a standard radiotracer incision assay. All of the selected inhibitors, except those possessing the highest IC_50_ values (i.e. PPNDS tetrasodium, cephapirin sodium and ceftriaxone sodium), yielded dose-dependent inhibition of APE1 in the gel-based assay, validating the present screening approach and indicating that the TAMRA/BHQ-2 assay is relatively insensitive to false-positive compounds acting via fluorescence interference.

As a step towards determining the biological potential of the top, validated APE1 inhibitors from the profiling assays above, we explored the ability of 6-hydroxy-DL-DOPA, Reactive Blue 2, myricetin, Tyrphostin AG 538, thiolactomycin, methyl 3,4-dephostatin and NSC-13755 to inactivate AP site cleavage activity of protein extracts from HEK 293T and HeLa cells. Of these compounds, 6-hydroxy-DL-DOPA, Reactive Blue 2 and myricetin had the most pronounced effect, leading to a significant reduction in total AP site cleavage activity, even amidst the pool of non-specific proteins. NSC-13755 also displayed potent inhibitory potential, but had been shown to fail in cell-based experiments [15]. The known bioactives Reactive Blue 2, 6-hydroxy-DL-DOPA and myricetin were subsequently demonstrated to enhance the cytotoxic and genotoxic potential of the alkylating agent MMS in cell culture assays, indicating specificity for APE1 and exemplifying their prospect as biological probes for APE1 function.

While our manuscript was under preparation, a report came out describing the identification of APE1 inhibitors using a virtual screen with a set of three-dimensional pharmacophore models generated based on key interactions of abasic DNA with the enzyme active site [45]. Notably, the inhibitors uncovered shared a few common features, including the requirement of at least one negatively ionizable (NI) group; the most potent inhibitors possessed two such groups separated by a hydrophobic core. Several hits identified in our screen are compatible with these findings. Among them is ATA, which contains dicarboxylates in close proximity similar to the more potent inhibitors reported [45], as well as a third carboxylate group that may augment the polar interactions within the APE1 active site; the diphenylmethylene core of ATA may occupy the hydrophobic pocket of the protein, as outlined in the authors' model [45]. In addition, 6-hydroxy-DL-DOPA, and the weaker inhibitors cephapirin and ceftriaxone, contain a single carboxylate, along with a relatively hydrophobic core decorated with various H-bond acceptor or donor combinations.

However, a consequence of utilizing interactions of abasic DNA with key APE1 active site residues to build the pharmacophore models is the potential to bias the results of the virtual compound database search. In particular, most of the models yielded compounds containing at least one carboxylate or bioisoteres that mimicked the NI group found in the phosphodiester backbone of DNA [45]. Their success in retrieving APE1 inhibitors led to the conclusion that design of potent, therapeutically relevant inhibitors must contain the features discussed above [45]. Yet, our screen of a diverse set of pharmacologically known actives unveiled more structurally diverse and potent inhibitors that do not appear to fit the pharmacophore models. An example is thiolactomycin, which did not share any of the “required” features. Furthermore, the strong effect observed with Reactive Blue 2, which contains no carboxylates, but instead possesses three readily ionizable sulfonate moieties, two of which are separated by a hydrophobic stretch, indicates that the requirement for a carboxyl substituent is not absolute. Although carboxylate containing compounds are likely to be prevalent among APE1 inhibitors, our screening results suggest that alternate interactions in the binding site may provide additional opportunities for the design of potent and selective endonuclease inhibitors. An example of this is 6-hydroxy-DL-DOPA, for which our modeling studies indicate that significant pi stacking interactions can occur between a ligand and the protein's sugar phosphate binding pocket. Such an interaction mode is different from the pharmacophore model developed by Zawahir et al [45], indicating a possibly new guiding principle for the design of small molecule inhibitors of APE1.

The most effective APE1 inhibitors within, i.e. Reactive Blue 2, 6-hydroxy-DL-DOPA and myricetin, were identified from the LOPAC^1280^, a collection of 1280 bioactive compounds representing 56 pharmacological classes. Such results point to APE1 as a novel target for these biomolecules and substantiate this repair endonuclease as a pharmacological target going forward. Reactive Blue 2 and its analogues are known to occupy the nucleotide-binding sites of a variety of proteins [46], and Reactive Blue 2 has been documented to be a selective antagonist of certain subtypes of P2Y receptors [47], [48]. It is possible that the inhibitory effect of Reactive Blue 2 on APE1 occurs via a similar active site occupancy mechanism [49], consistent with the recent report that free nucleotides (e.g. ATP) can regulate APE1 endonuclease efficiency [28]. 6-hydroxy-DL-DOPA is a precursor of the catecholaminergic neurotoxin 6-hydroxydopamine, and some of its reported neurotoxic effects may arise due to the inhibition of APE1 repair function [50]. Myricetin is a major flavonol, naturally occurring in a variety of vegetables, fruits and berries, as well as in beverages such as tea and wine [51], [52]. Myricetin exhibits several pharmacological benefits, and its antioxidant properties are thought to contribute to its cancer-preventive effects. However, myricetin has also been shown to induce DNA damage and promote mutagenesis in the Ames Test [53]. Myricetin appears to have several molecular targets, including thioredoxin reductase [54], mitogen-activated protein kinase kinase MEK1 [55], enzymes involved in the redox metabolism of polycyclic aromatic hydrocarbons [56], DNA and RNA polymerases, and in some instances topoisomerases [52], and the present study adds APE1 to this list.

## Supporting Information

Figure S1 (0.27 MB TIF)

Figure S2 (0.31 MB TIF)

Figure S3 (0.29 MB TIF)
