# Supplementary figures and images for: Identification and Characterization of Inhibitors of Human Apurinic/apyrimidinic Endonuclease APE1
Source: PLoS One. 2009 Jun 1;4(6):e5740. doi: 10.1371/journal.pone.0005740 (PMC2685009; doi:10.1371/journal.pone.0005740)

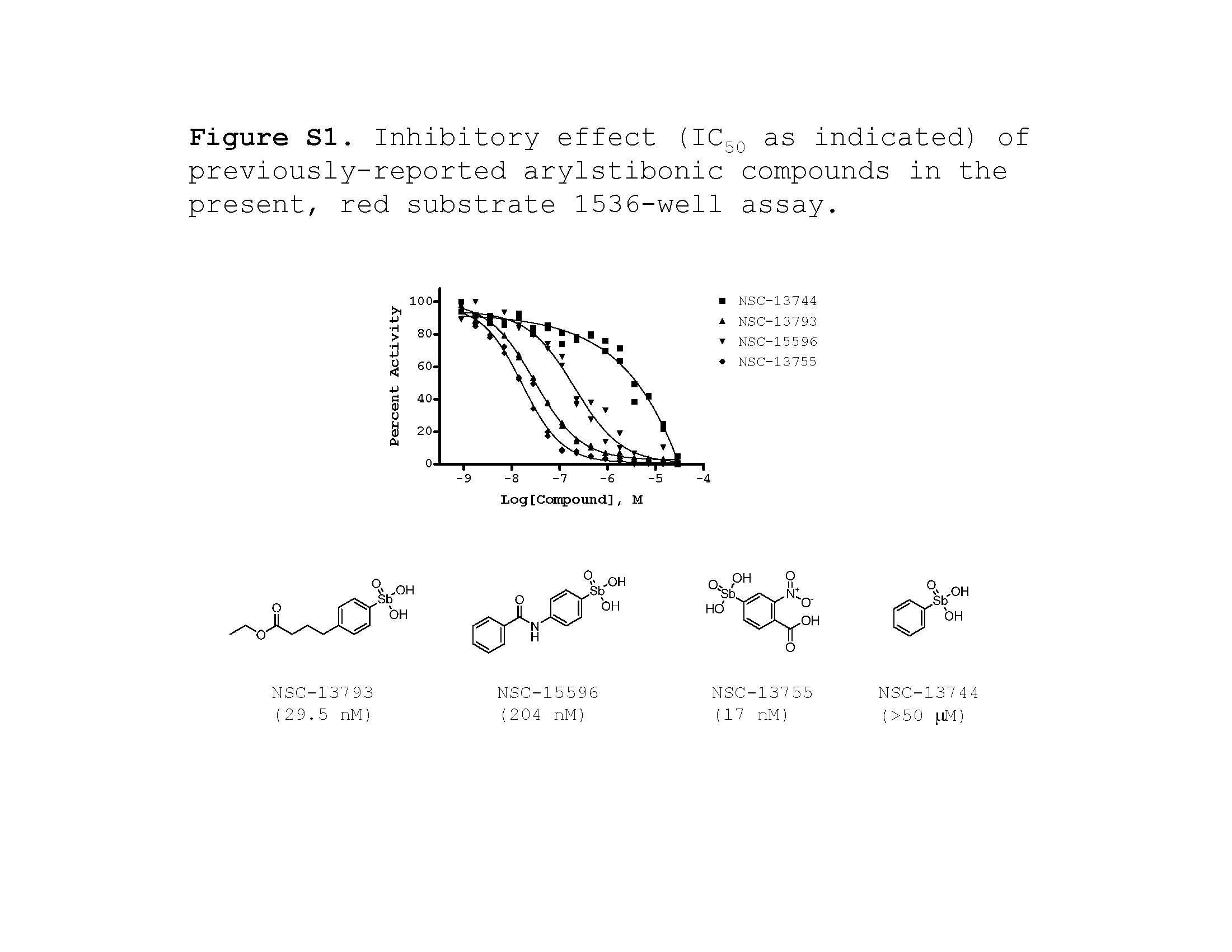

Supplement: Figure S1 — (0.27 MB TIF) [file pone.0005740.s001.tif]

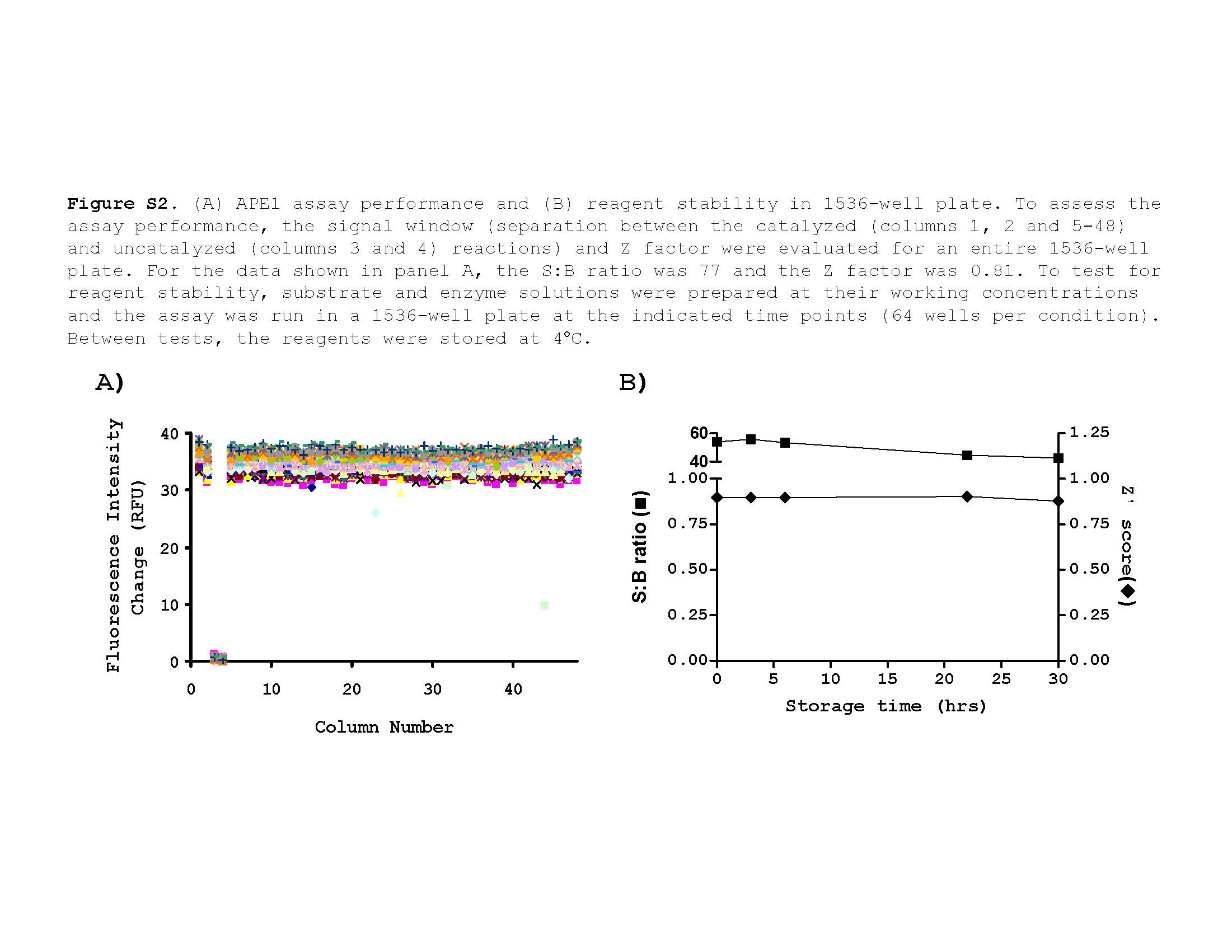

Supplement: Figure S2 — (0.31 MB TIF) [file pone.0005740.s002.tif]

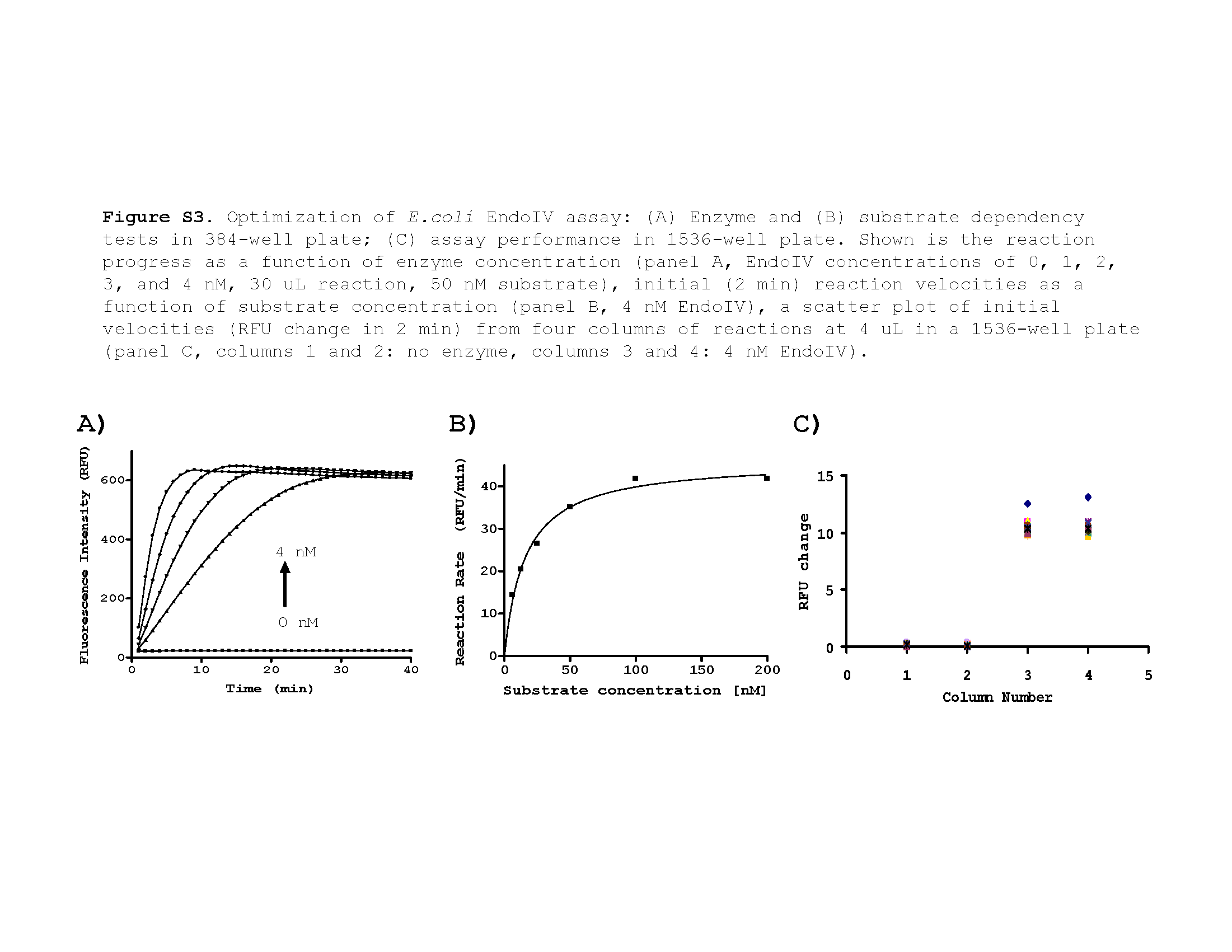

Supplement: Figure S3 — (0.29 MB TIF) [file pone.0005740.s003.tif]
